# Investigation of piperazines as human carbonic anhydrase I, II, IV and VII activators

**DOI:** 10.1080/14756366.2017.1417277

**Published:** 2017-12-27

**Authors:** Andrea Angeli, Niccolò Chiaramonte, Dina Manetti, Maria Novella Romanelli, Claudiu T. Supuran

**Affiliations:** Dipartimento Neurofarba, Università degli Studi di Firenze, Sezione di Scienze Farmaceutiche e Nutraceutiche, Sesto Fiorentino (Florence), Italy

**Keywords:** Carbonic anhydrase, activator, amine, piperazine, proton transfer, cognition enhancement

## Abstract

Four human (h) carbonic anhydrase isoforms (CA, EC 4.2.1.1), hCA I, II, IV, and VII, were investigated for their activation profile with piperazines belonging to various classes, such as N-aryl-, N-alkyl-, N-acyl-piperazines as well as 2,4-disubstituted derivatives. As the activation mechanism involves participation of the activator in the proton shuttling between the zinc-coordinated water molecule and the external milieu, these derivatives possessing diverse basicity and different scaffolds were appropriate for being investigated as CA activators (CAAs). Most of these derivatives showed CA activating properties against hCA I, II, and VII (cytosolic isoforms) but were devoid of activity against the membrane-associated hCA IV. For hCA I, the *K*_A_s were in the range of 32.6–131 µM; for hCA II of 16.2–116 µM, and for hCA VII of 17.1–131 µM. The structure-activity relationship was intricate and not easy to rationalize, but the most effective activators were 1-(2-piperidinyl)-piperazine (*K*_A_ of 16.2 µM for hCA II), 2-benzyl-piperazine (*K*_A_ of 17.1 µM for hCA VII), and 1-(3-benzylpiperazin-1-yl)propan-1-one (*K*_A_ of 32.6 µM for hCA I). As CAAs may have interesting pharmacologic applications in cognition and for artificial tissue engineering, investigation of new classes of activators may be crucial for this relatively new research field.

## 1. Introduction

Carbonic anhydrases (CAs, EC 4.2.1.1) are widespread metalloenzymes involved in the equilibration of carbon dioxide and bicarbonate, with formation of a proton^1–5^. This process can be described schematically by considering Equations (1 and 2), the first being the interconversion step between CO_2_ and bicarbonate, and the second one, which is rate-determining for the entire catalytic cycle, regenerates the nucleophilic, zinc hydroxide species of the enzyme^6–9^:

(1) $$H_{2}O\;EZn^{2+}-OH^{-}+CO_{2}\;⇆\;EZn^{2+}-{\text{HCO}}_{3}^{-}\;⇆\;EZn^{2+}-OH_{2}+{\text{HCO}}_{3}^{-}$$

(2) $$EZn^{2+}-OH_{2}\;⇆\;EZn^{2+}-\;HO^{-}+H^{+}$$

For this step to occur efficiently, a proton transfer reaction must take place from the Zn(II)-bound water molecule to the external medium (Equation (2)). Generally, this process is assisted by active site amino acid residues acting as proton shuttles, for example, His residues placed in the middle or at the entrance of the active site cavity^9^. In many human (h) CA isoforms, such as hCA II, IV, IX, XII, etc., this role of proton shuttle is played by His64^9^, but the possibility that a cluster of His residues (comprising residues 3, 4, 10, 15, and 64, hCA I numbering system) perform the shuttling has also been contemplated^10^, which may explain the fact that isoforms in which the cluster is present, such as hCA II and IX are among the most effective catalysts known in Nature^1^^,^^10^.

(3) $$EZn^{2+}-OH_{2}+A\;⇆\;[EZn^{2+}-OH_{2}-A]\;⇆[EZn^{2+}-OH^{-}-AH^{+}]\;⇆\;EZn^{2+}-{HO}^{-}+AH^{+}$$

Enzyme–activator complexes

**Figure 1.**
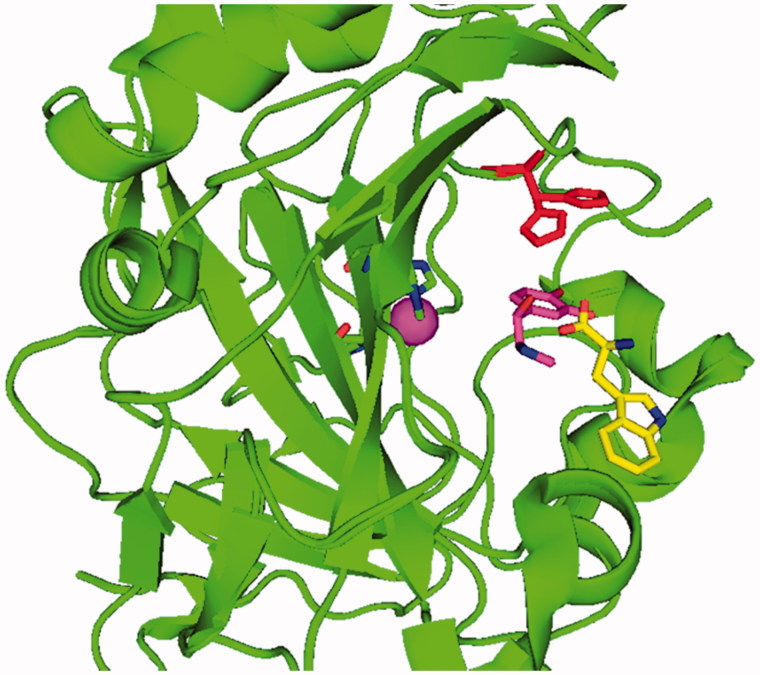
Complex of hCA II (green, ribbon diagram, Zn(II) ion as violet sphere) with L-adrenaline, in magenta, PDB file 2HKK^14^ and d-Trp, in yellow, PDB file 3EFI^13b^ in the activator binding sites A and B, respectively. His64, in red, is shown both in its “in” and “out” conformations. The two structures were superimposed.

**Figure 2.**
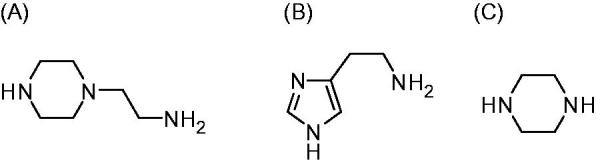
4-Aminoethyl-piperazine. **A**: histamine; **B:** piperazine; **C**: used as leads for the CAAs investigated in this paper.

It has been shown mainly by one of our groups^10^ that endogenous compounds able to participate in proton shuttling processes, in a similar manner to His64, act as CA activators (CAAs), by a mechanism described in Equation (3). The activator (A in Equation (3)) binds within the enzyme active site with formation of enzyme–activator complexes^7^, in which the activator molecule participates to the rate-determining step of the catalytic cycle, i.e. the proton shuttling from the water molecule coordinated to zinc to the external medium. Kinetic data in the presence of CAAs demonstrated that the activator does not influence *K*_M_ (the affinity for the substrate) and has an effect only on *K*_cat_ of the enzyme-catalyzed reaction, both for the esterase and CO_2_ hydrase activities of various CA isoforms^10–15^. X-ray crystallography of CA–activator complexes, such as the histamine, noradrenaline, l-/d-His, l-/d-Phe or d-Trp bound to hCA I and hCA II confirmed that the activators bind indeed at the entrance of the active site, not far away from His64 (which is present in two conformation, the “in” conformation – which is at around 6 Å from Zn(II), and the conformation pointing towards the exit of the active site, the “out” conformation – at >8 Å from Zn(II) as shown in Figure 1)^11^. As seen from Figure 1, the activator binding site is indeed far away from the metal ion, in the middle part of the active site, extending towards the rim of the cavity, in a region also occupied by His64 with its two different (in and out) orientations (Figure 2)^10^^,^^12–15^.

There are, in fact, two slightly different activator binding sites in α-CAs. Most activators except d-Trp^13^b bind in the activator binding site A (shown for l-adrenaline in Figure 1), whereas d-Trp is bound in an outer binding site compared to the other activators, denominated by the activator binding site B (Figure 1)^10^^,^^12–15^.

**Figure 3.**
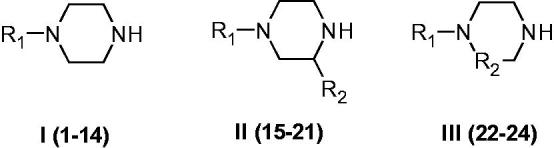
Piperazines **1–24** included in the CA activation study. For the meaning of R_1_ and R_2_ see Table 1.

There are thirteen catalytically active mammalian CAs, CA I-VA, VB, VI, VII, IX, XII-XV^1–3^. Apart from CA XV, which is not found in primates^1^, the remaining ones, CA I-XIV, are found in humans together with the murine (m) CA XV, they were investigated for their interaction with many activators, such as amino acids and amines^16–19^. Among them, 4-aminoethyl-piperazine **A**, which is structurally similar to histamine **B** (the first CAA investigated in detail, Figure 2) was an effective, low micromolar activator for several CA isoforms, but this was the only piperazine derivative investigated so far for such an activity^16–19^. Thus, in this paper, we report an activation study against four CA isoforms, hCA I, II, IV, and VII, with a library of piperazines incorporating a variety of scaffolds (Figure 3).

## 2. Experimental

### 2.1. Chemistry

Compounds **1–24** were either commercially, highest purity available derivatives from Sigma–Aldrich (Milan, Italy) and were used without further purification, or were prepared as described in the literature^20^.

### 2.2. Carbonic anhydrase assay

A stopped-flow method^21^ has been used for assaying the CA catalysed CO_2_ hydration activity with Phenol red as indicator, working at the absorbance maximum of 557 nm, following the initial rates of the CA-catalyzed CO_2_ hydration reaction for 10–100 s. For each activator, at least six traces of the initial 5–10% of the reaction have been used for determining the initial velocity. The uncatalyzed rates were determined in the same manner and subtracted from the total observed rates. Stock solutions of activator (0.1 mM) were prepared in distilled-deionized water and dilutions up to 0.1 nM were done thereafter with the assay buffer. The activation constant (*K*_A_), defined similarly with the inhibition constant *K*_I_, was obtained by considering the classical Michaelis–Menten equation (Equation (4)), which has been fitted by non-linear least squares by using PRISM 3:

(4) $$v=v_{\text{max}}/\{\text{1}+K_{\text{M}}/\left[\text{S}\right]\;\left(\text{1}+{\left[\text{A}\right]}_{\text{f}}/K_{\text{A}}\right)\}$$

where [A]_f_ is the free concentration of activator.

Working at substrate concentrations considerably lower than *K*_M_ ([S] ≪ *K*_M_), and considering that [A]_f_ can be represented in the form of the total concentration of the enzyme ([E]_t_) and activator ([A]_t_), the obtained competitive steady-state equation for determining the activation constant is given by Equation (5)^22–26^:

(5) $$v\;=\;v_{0}.K_{\text{A}}/\{K_{\text{A}}\;+\;({\left[\text{A}\right]}_{\text{t}}\;–\;0.\text{5}{\left\{\left({\left[\text{A}\right]}_{\text{t}}\;+\;{\left[\text{E}\right]}_{\text{t}}\;+\;K_{\text{A}}\right)\;–\;{\left({\left[\text{A}\right]}_{\text{t}}\;+\;{\left[\text{E}\right]}_{\text{t}}\;+\;K_{\text{A}}\right)}^{\text{2}}\;–\;\text{4}{\left[\text{A}\right]}_{\text{t}}.\left[\text{E}\right]\text{t}\right)}^{\text{1}/\text{2}}\}\}$$

where *v*_0_ represents the initial velocity of the enzyme-catalyzed reaction in the absence of an activator. All CA isozymes used in the experiments were purified recombinant proteins obtained as reported earlier by our group^22–26^.

## 3. Results and discussion

### 3.1. Chemistry

Piperazines **1–24** (Figure 3) were chosen to be investigated as CAAs as they contain the endocyclic NH group able to participate in proton shuttling processes between the zinc-coordinated water from the CA active site and the external medium, in a similar manner to 4-aminoethyl-piperazine **A**, and histamine **B**, which were considered as lead compounds. Furthermore, in contrast to **A** and **B**, piperazines **1–24** do not possess the aminoethyl moiety present in the two leads, but the p*K*_a_ of the NH (or NR) groups from the heterocyclic ring is influenced by the diverse substitution patterns present in them. Indeed, both electron withdrawing as well as electron donating moieties are present in these compounds which may lead to a different basicity of the moieties able to shuttle protons between the enzyme active site and the reaction medium (Figure 3). The unsubstituted piperazine (compound **C**) was also tested for comparison.

### 3.2. CA activation

Activation data against four physiologically relevant hCA isoforms, hCA I, II, IV, and VII, are shown in Table 1. Indeed, hCA I, II, and IV are involved in a multitude of eye diseases^1^^,^^27^, and their inhibition is pharmacologically used for the treatment of glaucoma^27^, edema^28^, obesity^29^, and hypoxic tumors^30^, whereas more recently, some of these isoforms were also validated as drug targets for neuropathic pain^31^, cerebral ischemia^32^, and arthritis^33^. Thus, modulators, potentially with selective action, both for inhibiting and activating these enzymes, are of great pharmacological interest. In fact, recently CAAs were shown to potentiate cognition through the phosphorylation of the extracellular signal-regulated kinase in the cortex and the hippocampus of model animals^34^, being thus of great interest for memory therapy. Furthermore, Muller’s group showed that CAAs potentiate the initial steps of bone formation in models of artificial mineralization processes^35^.

**Table 1.** CA activation of isoforms hCA I, II, and VII (cytosolic) and IV (membrane-associated) with compounds **1-24**, by a stopped-flow CO_2_ hydrase assay^21^.
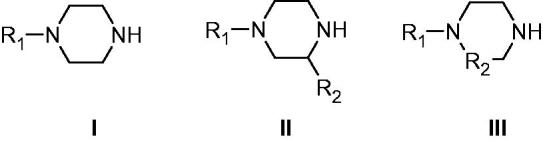

				*K*Aa (µM)			
Compound	Structure	R_1_	R_2_	hCA I	hCA II	hCA IV	hCA VII
**A**^b^	I	CH_2_CH_2_NH_2_	–	7.41	2.30	24.9	32.5
**B**^b^	–	–	–	2.10	125.0	25.3	37.5
**C**	I	H	–	>150	>150	>150	>150
**1**	I	Phenyl	–	>150	74.9	>150	121.3
**2**	I	4-F-phenyl	–	88.2	38.7	>150	47.8
**3**	I	4-Cl-phenyl	–	104.0	110.3	>150	126.1
**4**	I	4-MeO-phenyl	–	48.6	50.1	>150	80.4
**5**	I	4-COMe-phenyl	–	83.7	97.9	>150	>150
**6**	I	3-Cl-phenyl	–	95.2	82.7	>150	104.0
**7**	I	3-MeO-phenyl	–	119.2	80.1	>150	>150
**8**	I	3-CF_3_-phenyl	–	110.2	77.6	>150	114.5
**9**	I	2-Pyridyl	–	131.0	75.2	>150	95.2
**10**	I	Methyl	–	>150	78.4	>150	97.0
**11**	I	Benzyl	–	>150	85.3	>150	98.4
**12**	I	Acetyl	–	127.4	109.0	>150	96.4
**13**	I	CH_2_CH_2_OH	–	102.1	91.6	>150	124.2
**14**	I	2-Piperidinyl	–	62.5	16.2	>150	49.2
**15**	II	H	Methyl	>150	84.0	>150	131
**16**^c^	II	H	Phenyl	80.3	49.7	>150	>150
**17**^c^	II	Benzoyl	Phenyl	75.2	84.5	>150	35.2
**18**^c^	II	H	Benzyl	73.7	116	>150	17.1
**19**^c^	II	Propionyl	Benzyl	32.6	36.1	>150	84.0
**20**^c^	II	Benzoyl	Benzyl	85.2	82.4	>150	48.5
**21**	II	H	COOH	47.9	46.8	>150	93.6
**22**	III	H	CO	115.0	>150	>150	37.1
**23**	III	H	CH_2_CH_2_	79.4	44.6	>150	98.5
**24**	III	Benzyl	CH_2_CH_2_	48.1	33.2	>150	127

Aminoethylpiperazine **A** and histamine **B** were used as standard activators.

a Errors in the range of ±5–10% of the reported values (data not shown) from three different assays.

b Data for **A,B** from Vullo et al.^18^.

c Prepared as described in Guandalini et al.^20^.

The activation of the four CA isoforms mentioned above with the piperazine derivatives **1–24** and two standard activators (compounds **A** and **B**) shown in Table 1 allowed us to delineate the following structure-activity relationship (SAR):

- Although unsubstituted piperazine C was inactive as a CAA (*K*_A_s > 150 µM against all investigated enzymes), the substituted-piperazines **1–24** showed CA activating properties against hCA I (except compounds **1, 10**, **11,** and **15**, which had *K*_A_s > 150 µM) with activation constants ranging between 32.6 and 131 µM, being thus moderate – weak activators. Indeed, the leads **A** and **B** were much more potent, low micromolar activators of this isoform, with *K*_A_s of 2.1–7.4 µM (Table 1)^18^. The best hCA I activators in the series of investigated compounds were **4, 19, 21,** and **24** (*K*_A_s of 32.6–48.1 µM), and they belong to variously substituted piperazines. Small variations on the core structure of these compounds generally led to a diminution of the activity. For example, **19**, the best hCA I activator, carries a propionyl group on the piperazine ring and a benzyl moiety in the 3 position. Its deacylated analog, **18**, was almost two times a less effective hCA I activator, with a *K*_A_ of 73.7 µM, compared to **19**.
- The physiologically dominant cytosolic isoform hCA II was more sensitive to activation with piperazines **1–24** investigated here compared to hCA I (Table 1). Thus, only **22** was inactive (*K*_A_ > 150 µM), and the range of the activation constants for the remaining derivatives was of 16.2–116 µM. A number of compounds showed *K*_A_s in the range of 16.2–50.1 µM: for example, **2, 4, 14, 19, 21, 23,** and **24**. They belong to various chemical classes and incorporated different substituents, which demonstrates that it might be possible to design much more efficient CAAs incorporating this interesting ring. However, the simple lead compound **A** was a much more potent hCA II activator compared to the other piperazines investigated here, whereas histamine **B** was a very inefficient hCA II activator with a *K*_A_ of 125 µM (Table 1). Amazingly, the best hCA II activator was **14**, which has two potential piperidine rings that may participate in the proton shuttling processes.
- Surprisingly, the membrane-bound isoform hCA has not activated significantly by any of the piperazines investigated here, although the leads **A** and **B** showed medium potency efficacy with *K*_A_s of 24.9–25.3 µM.
- The brain cytosolic isoform hCA VII was not activated by piperazines **5**, **7,** and **16** (*K*_A_ > 150 µM), whereas the remaining derivatives showed a profile of medium – weak activator, with *K*_A_s in the range of 17.1–131 µM (Table 1). The best hCA VII activators were **2, 17, 18, 20,** and **22** (*K*_A_s in the range of 17.1–48.5 µM). For this isoform, the SAR of the couple **18/19** is completely different compared to what is mentioned above for the activation of hCA I. In this case, the deacetylatyed derivative **18** was 4.9 times a better hCA VII activator compared to the acetylated one **19**. Thus, small changes in the scaffold lead to a very different activation profile in this series of piperazines and their derivatives.

## 4. Conclusions

We report here an activation study of four physiologically and pharmacologically relevant CA isoforms, hCA I, II, IV, and VII with a rather large series of piperazines and their derivatives. The compounds were included in order to investigate whether the fine tuning of the basicity correlated with the various shapes of these molecules may lead to efficient activators, considering the fact that the only piperazine investigated till now as activator (4-(2-aminoethy)l-piperazine) showed such interesting properties. hCA I was activated by most of the investigated derivatives, with activation constants of 32.6–131 µM; hCA II with activation constants of 16.2–116 µM, whereas the membrane-bound isoform hCA IV was not activated by the investigated piperidines. The brain-associated cytosolic isoform hCA VII was activated with *K*_A_s in the range of 17.1–131 µM. The structure-activity relationship was intricate and not easy to rationalize for each isoform, but the most effective activators were 1-(2-piperidinyl)-piperazine **14** (*K*_A_ of 16.2 µM for hCA II), 2-benzyl-piperazine **18** (*K*_A_ of 16.2 µM for hCA VII), and 1-(3-benzylpiperazin-1-yl)propan-1-one **19** (*K*_A_ of 32.6 µM for hCA I). As CAAs may have interesting pharmacologic applications in cognition and for artificial tissue engineering, investigation of new classes of activators as the ones reported here may be crucial for this relatively new research field.
